# Unexpectedly High Prevalence of Common Variable Immunodeficiency in Finland

**DOI:** 10.3389/fimmu.2017.01190

**Published:** 2017-09-28

**Authors:** Jannica S. Selenius, Timi Martelius, Sampsa Pikkarainen, Sanna Siitonen, Eero Mattila, Risto Pietikäinen, Pekka Suomalainen, Arja H. Aalto, Janna Saarela, Elisabet Einarsdottir, Asko Järvinen, Martti Färkkilä, Juha Kere, Mikko Seppänen

**Affiliations:** ^1^Folkhälsan Institute of Genetics, Helsinki, Finland; ^2^Adult Immunodeficiency Unit, Infectious Diseases, Inflammation Center, University of Helsinki, Helsinki University Hospital, Helsinki, Finland; ^3^Department of Gastroenterology, University of Helsinki, Helsinki University Hospital, Helsinki, Finland; ^4^Laboratory Services, Hospital District of Helsinki and Uusimaa Laboratory, University of Helsinki, Helsinki University Hospital, Helsinki, Finland; ^5^Department of Infectious Diseases, Kymenlaakso Central Hospital, Kotka, Finland; ^6^Department of Infectious Diseases, South Karelia Central Hospital, South Karelia Social and Health Care District, Lappeenranta, Finland; ^7^Department of Medicine, Kuopio University Hospital, Kuopio, Finland; ^8^Institute for Molecular Medicine Finland (FIMM), University of Helsinki, Helsinki, Finland; ^9^Department of Biosciences and Nutrition, Karolinska Institutet, Huddinge, Sweden; ^10^Molecular Neurology Research Program, University of Helsinki, Helsinki, Finland; ^11^Department of Medical and Molecular Genetics, King’s College London, London, United Kingdom; ^12^Rare Disease Center, Children’s Hospital, University of Helsinki, Helsinki University Hospital, Helsinki, Finland

**Keywords:** primary immunodeficiency, common variable immunodeficiency, prevalence, primary antibody deficiency, secondary antibody deficiency, hypogammaglobulinemia

## Abstract

**Background:**

Common variable immunodeficiency (CVID) is the most common primary immunodeficiency. Prevalence varies greatly between countries and studies. Most diagnostic criteria include hypogammaglobulinemia and impaired vaccine response.

**Aim:**

To evaluate the minimum prevalence as well as the clinical and immunological phenotypes of CVID in Southern Finland.

**Methods:**

We performed a cross-sectional study to assess all adult CVID patients followed up in three hospital districts in Southern and South-Eastern Finland between April 2007 and August 2015. CVID diagnosis was based, with a minor modification, on the ESID/PAGID criteria for primary CVID. Antipolysaccharide responses to Pneumovax^®^ were defined as impaired only if 50% or more of the serotypes did not reach a level of 0.35 µg/mL after vaccination. We further characterized the patients’ B cell phenotypes and complications associated with CVID.

**Results:**

In total, 9 patients were excluded due to potential secondary causes before diagnosis. ESID/PAGID criteria were met by 132 patients (males 52%), of whom, 106 had “probable” and 26 “possible CVID.” Based on the population statistics in the three hospital districts, the minimum adult prevalence per 100,000 inhabitants in Finland for all CVID (“probable CVID,” respectively) patients was 6.9 (5.5). In the highest prevalence district (Helsinki and Uusimaa), the prevalence was 7.7 (6.1). CVID patients suffer from frequent complications. Ten patients died during follow-up. Of probable CVID patients, 73% had more than one clinical phenotype. Intriguingly, gradual B cell loss from peripheral blood during follow-up was seen in as many as 16% of “probable CVID” patients. Patients with possible CVID displayed somewhat milder clinical and laboratory phenotypes than probable CVID patients. We also confirm that large granular lymphocyte lymphoproliferation is a CVID-associated complication.

**Conclusion:**

The prevalence of CVID in Finland appears the highest recorded, likely reflecting the genetic isolation and potential founder effects in the Finnish population. Studies to discover potential gene variants responsible for the high prevalence in Finland thus seem warranted. Increased awareness of CVID among physicians would likely lead to earlier diagnosis and improved quality of care.

## Introduction

Common variable immunodeficiencies (CVID) are jointly the most common clinically significant primary immunodeficiencies in the world. The prevalence varies greatly between countries, ranging from 3.8/100,000 in Denmark to 0.6/100,000 in Spain (Table 1) (1–8). There are also geographical differences in setting diagnosis. CVID has classically been diagnosed using the old ESID/PAGID criteria (9). In recent years, new criteria, such as the revised ESID Registry (10), Ameratunga (11), and International Consensus Document (ICON) (12) criteria suggest updated approaches to diagnosing CVID. The largest differences between countries in setting a diagnosis within these criteria lie in the interpretation of antipolysaccharide vaccine responses. Current practices and guidelines differ greatly between countries, with major variation in definitions due to small or unpublished control populations (13–15). The most stringent serotype-specific criteria suggested are based on population-derived data and advocate a threshold of 0.35 µg/mL for protectivity (15).

**Table 1:** Prevalence of CVID in various countries.

Country	Prevalence of CVID/100,000	% of CVID of total number of primary immunodeficiencies	Reference
Denmark^a^	3.8	n.a.	(8)
Iceland	3.1	17	(7)
Norway	2.6	31	(3)
Turkey	1.4	5	(4)
United Kingdom	1.3	37	(5)
Switzerland	1.2	45	(6)
France	0.7	14	(1)
Spain	0.6	21	(2)

*CVID, common variable immunodeficiency; n.a., not available*.

*^a^In two Danish regions, the prevalence was 5/100,000*.

There are no contemporary studies available on the prevalence of CVID in Finland (16). The aim of this study was to estimate the minimum adult (aged ≥16 years) prevalence of CVID in three hospital districts in Finland that jointly serve approximately 1.9 million inhabitants (i.e., ≈38% of the Finnish population). To delineate a universally accepted minimum prevalence of CVID in Finland, the cut-offs of impaired antipneumococcal polysaccharide responses to a non-conjugated 21-valent pneumococcal vaccine (Pneumovax^®^) were based on the stringent population-based data from population-based vaccine response studies (15). We studied the patients’ B cell and clinical phenotypes and compared the findings between “probable” and “possible CVID” patients (throughout the article, “probable” and “possible CVID” refer to ESID/PAGID criteria). Clinical phenotypes were defined by clinical complications associated with CVID (17).

## Materials and Methods

### Study Population

Patients were initially diagnosed with CVID in 1960–2015. CVID diagnosis was re-assessed in all follow-up patients with IgG substitution therapy. Historical patients without vaccine response data (*n* = 56) were considered to have CVID if at least two of the three main immunoglobulin classes (IgG, IgA, IgM) had been below reference without apparent secondary causes noted at diagnosis or during early follow-up. Between February 2007 and August 2015, patients with low levels of at least two of the three main immunoglobulin classes IgG, IgA, and IgM were immunologically assessed using previously published routine accredited laboratory methods (18).

Pneumococcal vaccine responses were assessed at the Finnish National Institute for Health and Welfare, as previously described (13, 15). Of patients with vaccine response data, the majority were tested for responses to seven serotypes (4, 6B, 9V, 14, 18C, 19F, 23F) (13) and the rest to 10 serotypes (1, 4, 5, 6B, 7F, 9V, 14, 18C, 19F, 23F) (15). Patients were defined to have poor responses to polysaccharide antibodies if at least 50% of the measured serotype-specific antipneumococcal polysaccharide antibody titers were below 0.35 µg/mL 4 weeks after vaccination with Pneumovax^®^ (15).

Participating outpatient clinics were the only ones providing adult ambulatory care for primary immunodeficiencies in these areas, exclusively authorized to assess reimbursement criteria for long-term IgG replacement therapy. All patients living within the Hospital District of Helsinki and Uusimaa and followed up at the Adult Immunodeficiency Unit of Helsinki University Hospital (HUH), as well as individuals in the respective districts and outpatient clinics in Kymenlaakso Social and Health Services (Carea) and South Karelia Social and Health Care District (Eksote) were systematically assessed for CVID using the above-listed inclusion criteria. Patients with CVID who visited HUH for consultation only from outside the study area were excluded from analyses. No data were available for patients younger than 16 years of age at the end of the study.

### CVID Criteria Used

We used the ESID/PAGID criteria to define CVID, with the exception that patients with impaired vaccine responses, normal IgG, and below reference concentration of plasma IgM and IgA were moved into the “possible CVID” category (9). We also required the clinical phenotype during follow-up to be typical for CVID. If in the historical cohort, a secondary cause was suspected or the patient tolerated IgG therapy poorly, patients were reevaluated with pneumococcal antibody response testing after a pause in IgG replacement therapy with a 4- to 6-month washout period. If they refused reassessment, they were excluded from the cohort. Pretreatment IgG levels for the historical cohort were 0.0–3.8 (median 1.4) g/L and below 3.8 for all. If a combined immunodeficiency was suspected clinically during follow-up, we excluded those with at least two of the following: low CD4 counts, low naïve CD4 cell counts, and/or absent T cell proliferation. Due to the long follow-up available, these were not systematically assessed without prior clinical suspicion in patients diagnosed >4 years before the end of study. Those fulfilling late-onset combined immunodeficiency (LOCID) criteria based on having a CD4^+^ T-cell count lower than 200 × 10^6^ cells/L on more than one occasion, without explainable cause, were retained in the cohort (19).

### Clinical Phenotyping

We assessed five clinical phenotypes (17). To enable comparisons between previously published cohorts, “autoimmunity” included the following inflammatory conditions: idiopathic thrombocytopenia purpura (ITP), autoimmune hemolytic anemia (AIHA), hyper- and hypothyroidism, diabetes mellitus type 1, vitiligo, psoriasis, alopecia, rheumatic arthritis, celiac disease, pernicious anemia, atrophic gastritis, autoimmune neutropenia, sialadenitis, and primary sclerosing cholangitis. “Polyclonal lymphoproliferation” included lymphoid interstitial pneumonia, lymphadenopathy, splenomegaly, granulomatous-lymphocytic interstitial lung disease (GLILD, proven with biopsy or typical radiology) and other, biopsy-proven granuloma (excluding Crohn’s disease). The other three phenotypes were “malignancies,” “non-infectious gastrointestinal disease,” and “infections only” (17). Due to the high variability of gastrointestinal complications in CVID patients, these were all listed under one subheading and will be described separately.

### Immunophenotyping

Lymphocyte and B cell phenotyping were performed as previously published (20). Briefly, for flow cytometry, cells in EDTA samples were stained using whole blood technique, and analyzed on a FACSCanto II flow cytometer [Beckton Dickinson (BD) Biosciences, San Jose, CA, USA]. To establish the relative and absolute numbers in each lymphocyte subset (CD3^+^ T cells, CD4^+^ T cells, CD8^+^ T cells, CD19^+^ B cells, and CD16^+^CD56^+^ NK cells), MultiTEST CD3 (clone SK7), FITC/CD8 (clone SK1), PE/CD45 (clone Hle-1), PerCP/CD4 (clone SK3), APC, and CD3 FITC/CD16 (clone B73.1) + CD56 (clone NCAM 16.2), PE/CD45 PerCP/CD19 (clone SJ25C1), APC reagents, and TruCOUNT Tubes (BD Biosciences) were used according to manufacturer’s instructions. To establish the relative numbers in B memory cell subsets, 100 µl of whole blood was washed three times with phosphate-buffered saline, followed by staining for 15 min at room temperature with the following antibodies at optimal concentrations: anti-IgD FITC (clone IA6-2), anti-IgM APC (clone G20-127), CD19 PE-Cy7 (clone SJ25C1), CD21 PE (clone B-ly4), CD27 PE (clone L128), CD38 PerCP-Cy5.5 (clone HIT2), and CD45 APC-Cy7 (clone 2D1) all from BD Biosciences. After lysis of red blood cells with the BD FACS Lysing Solution (BD Biosciences) and washing, data acquisition was performed on a FACSCanto II flow cytometer. A minimum of 5,000 CD19^+^ lymphocytes was acquired. Data were further analyzed using FACS Diva software version 6.1.3 (BD Biosciences), and B cell subsets determined according to Wehr et al. (20).

### Data Handling and Analyses

Coded data were collected into an online database created using Microsoft Access. The database held categories containing demographic information, laboratory results used in different classification criteria, clinical phenotypes, and infections. Those without data were excluded from sub-analyses.

The database had mandatory fields to ensure the quality of the study as well as pre-filled alternatives to choose from to minimize errors. Cytopenias were defined as neutropenia, lymphopenia, or thrombocytopenia below 2.5 centile for more than 3 months. Chronic or recurrent gastroenteritis was defined as ≥3 bacterial or viral gastroenteritis and/or chronic excretion of virus to feces over 3 months.

The robustness of CVID diagnosis was compared between the original ESID/PAGID (1999), the Ameratunga (2013), and the ICON (2015) criteria (9, 11, 12). No comparisons to the revised ESID registry criteria (2014) were performed due to its debatable exclusion of CVID-like patients with low levels of IgG and IgM but normal IgA and for the lack of systematic naïve CD4 cell and lymphocyte proliferation data in historical patients (12, 21).

Prevalence calculations were based on the reported population figures given by Statistics Finland (http://www.stat.fi/til/vrm_en.html) and on the known hospital district populations. Using the Access database, relationships between clinical manifestations and differences between patients in the hospital districts were analyzed.

Microsoft Excel and IBM SPSS Statistics 22.0 were used for the analysis. Frequencies and co-expression of CVID clinical complications were studied using descriptive the statistics/frequencies and crosstabs functions. For comparisons of continuous variables between groups, the Mann–Whitney *U*-test was used. *p*-Values <0.05 were considered significant.

### Ethics

The study was approved by the Coordinating Ethics Committee of the Helsinki and Uusimaa Hospital District (138/13/03/00/2013).

## Results

### Demographics

**Figure 1:**
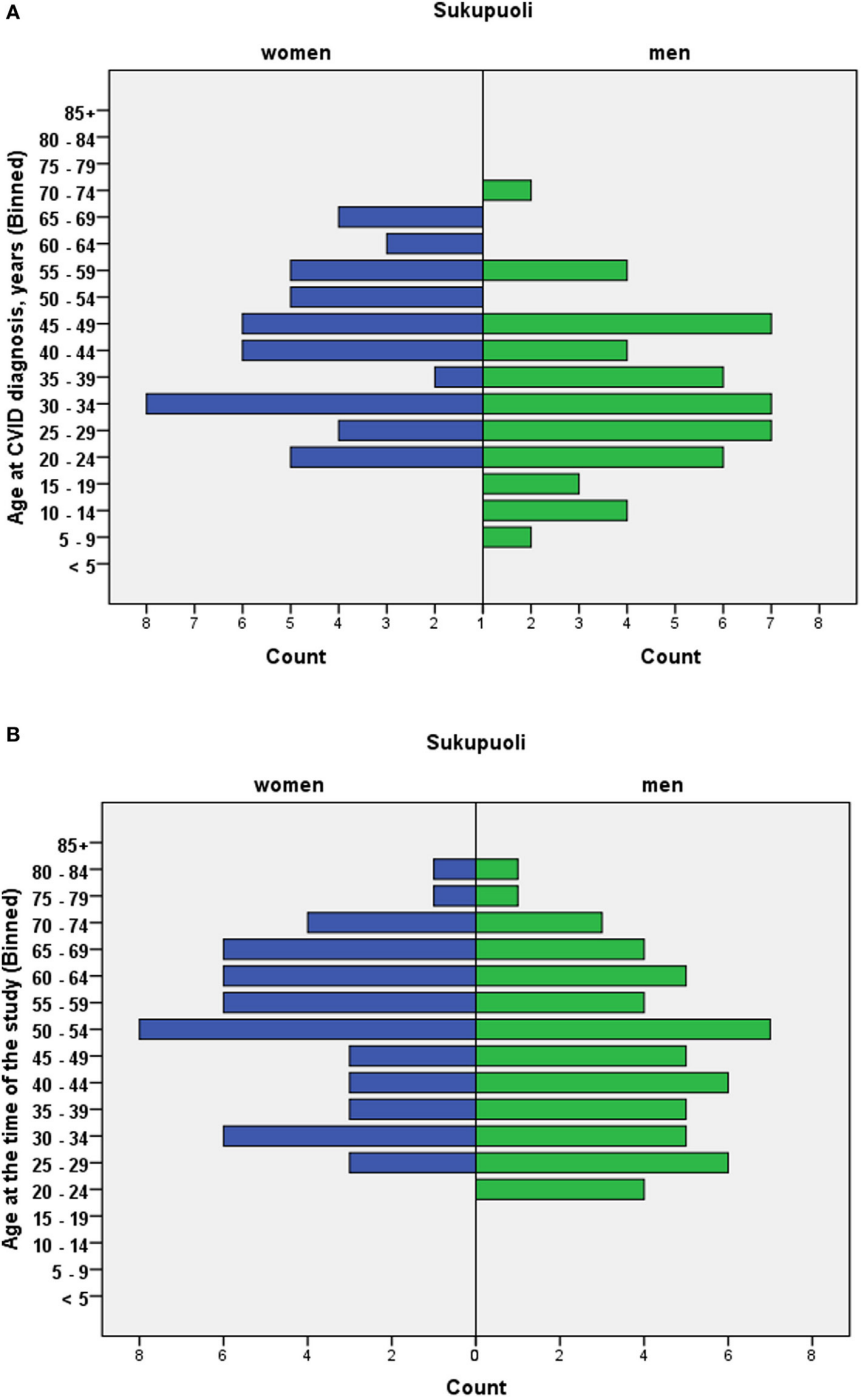
Age at diagnosis **(A)** and age at the time of the study **(B)**. 106 patients with “probable CVID.”

Of 141 patients, nine were considered to have secondary CVID (non-Hodgkin lymphoma 7, unclassified hematologic disease 2; 4 had received treatment with rituximab), and were not studied further. Of the remaining 132 patients with primary CVID, 106 patients were diagnosed with “probable CVID” and 26 patients with “possible CVID” according to the ESID/PAGID criteria. Of the 26 patients with “possible CVID,” 25 had low levels of IgG, with impaired antipneumococcal polysaccharide responses (specific antibody deficiency) and normal IgA and IgM levels. One previously published “possible CVID” patient with an *NFKB1* I553M mutation had normal IgG but low IgA and IgM levels as well as impaired antipneumococcal vaccine responses and would have been classified as “probable CVID” in the original ESID/PAGID criteria (22). Of the remaining 106, four patients were diagnosed with LOCID. The age of patients at diagnosis and at the time of the study is presented in Figures 1A,B. Of the 106 “probable CVID” patients, the male/female sex ratio was 1.1; ratios in different age groups (in years) were as follows: <30:4; 30–59:1.1; >59:0.78. The age range in years for the whole CVID cohort was 9–74 at diagnosis and 20–84 at the time of study. Of the nine CVID patients diagnosed before age 20, eight (89%) were male. Of the 29 CVID patients diagnosed before age 30, 20 (68%) were male. Ten patients died during the study, three due to gastrointestinal carcinomas, while there were single cases of myeloid sarcoma, subarachnoid hemorrhage, acute myocardial infarction, suspected substance intoxication; three remained unknown. The mean age of death was 61.3 years.

### Prevalence of CVID in Finland

We studied how prevalent patients in the studied hospital districts with “probable CVID” and “possible CVID” were according to ESID/PAGID criteria (Table 2). Based on available data, the minimum adult prevalence of CVID in Finland was 6.9/10,000 (5.5/10,000 for “probable CVID”), reaching 7.7/10,000 (6.1/10,000 for “probable CVID”) in the university teaching hospital district (HUS).

**Table 2:** Estimated minimum adult prevalence of “probable” and combined “probable” and “possible CVID” in the studied Finnish hospital districts according to 1999 ESID/PAGID criteria.

	Hospital District of Helsinki and Uusimaa (HUS)	Hospital District of Kymenlaakso (Carea)	Hospital District of South Carelia (Eksote)	Finland
Population (*n*)	1,616,300	171,000	131,155	5,500,000
Probable CVID	6.1	2.9	1.5	5.5
Possible CVID	1.6	0	0	1.4

*CVID, common variable immunodeficiency*.

### Comparisons between Different CVID Criteria

We compared ESID/PAGID, Ameratunga, and ICON criteria for setting the CVID diagnosis (Table 3) (9, 11, 12). When using the ESID/PAGID criteria, our study consisted of 106 “probable” and 26 “possible” CVID patients. The Ameratunga criteria yielded 109 probable and 23 possible CVID patients. ICON criteria only diagnose definite CVID, and our study had 63 such patients. However, if all the historical patients lacking vaccine response tests who otherwise fulfill the ICON criteria were included, the number of definite CVID patients in the whole study area would have been 113 (prevalence 5.9/100,000).

**Table 3:** Numbers of patients with CVID according to three diagnostic criteria.

	ESID/PAGID	Ameratunga	ICON
Probable CVID	106	109	–
Possible CVID	26	23	–
Definite CVID	0	0	63 (113^a^)

*ESID, European Society for Immunodeficiencies; PAGID, Pan-American Group for Primary Immunodeficiencies; ICON, International Consensus Document*.

*^a^When historical patients, who otherwise fulfill ICON criteria, but lack vaccine response data, have been added into the total count*.

### Clinical Manifestations

**Figure 2:**
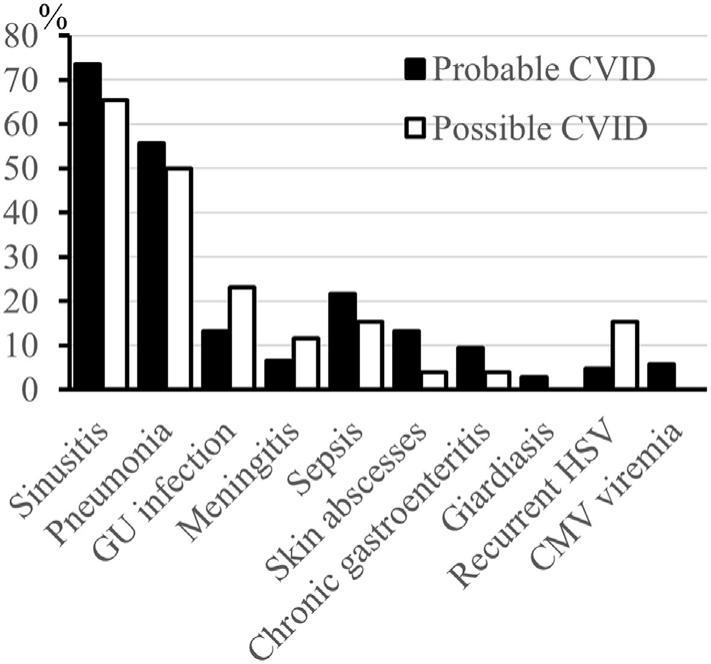
Infections (%) in probable and possible CVID patients. No statistical differences were noted between “probable” and “possible CVID” patients. GU, genitourinary. Sepsis includes patients with a severe bacteremic infection leading to hospitalization.

**Figure 3:**
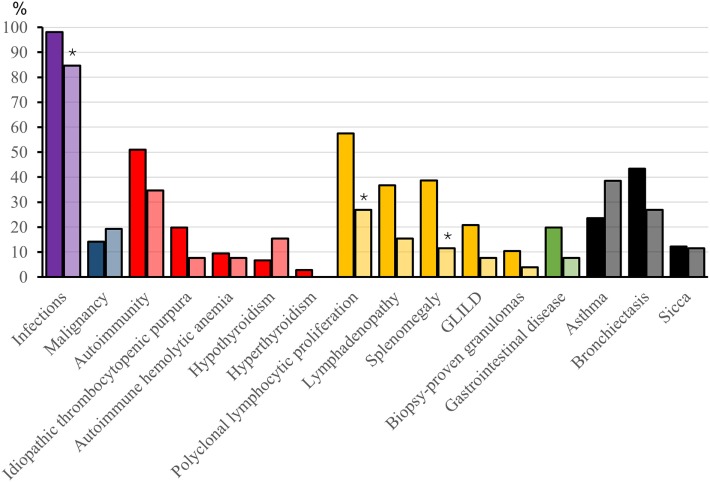
Complications (%) in probable and possible CVID patients. Asterisks mark statistical significance between “probable” and “possible CVID” patients, *p < 0.05 (Fisher’s 2-sided Exact test or Pearson Chi-square, as appropriate). For each complication, data are presented with pairwise bars for “probable” and “possible CVID” where “probable CVID” is first and with darker color. Sicca diagnosis was based on doctor-assessed symptoms and findings.

**Figure 4:**
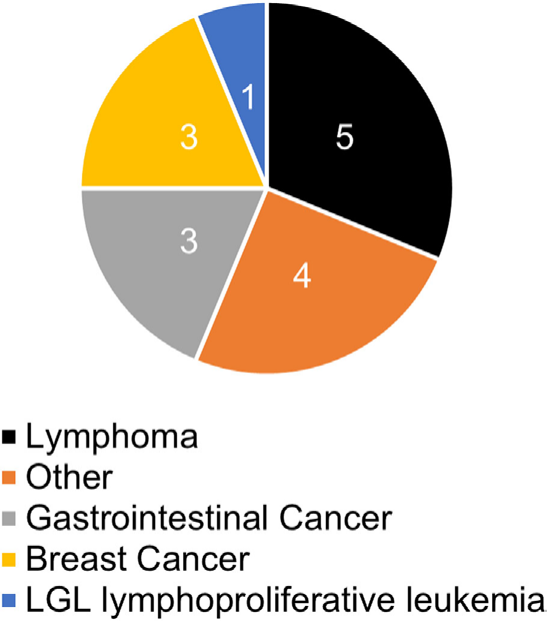
Malignancies (%) in probable CVID patients. Other four cancers include one cervical cancer and one renal carcinoma, one melanoma, and one myeloid sarcoma. LGL, large granular lymphocytic.

**Figure 5:**
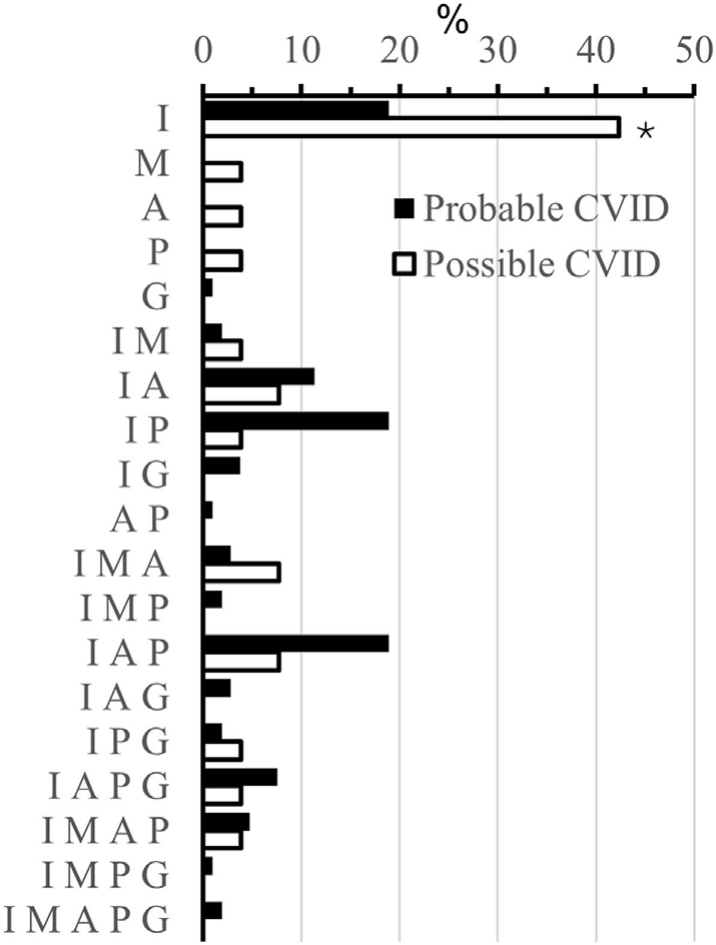
Different phenotypes (%) in probable and possible CVID. Phenotypes are abbreviated as follows; also their combinations are used: I, infections; M, malignancies; A, autoimmunity; P, polyclonal lymphocytic proliferation; G, gastrointestinal disease. Asterisks mark statistical significance between “probable” and “possible CVID” patients, *p < 0.05 (Fisher’s 2-sided Exact test).

A history of infections was noted in 104 (98%) “probable CVID” patients. The most frequent significant infections were pneumonia, sinusitis, sepsis, genitourinary infections, and skin abscesses (Figure 2). Associated clinical manifestations are shown in Figure 3. Within the cohort of “probable CVID” patients, 54 (51%) patients suffered from at least one autoimmune disorder. The most common autoimmune manifestations were ITP and AIHA (Figure 3). Altogether, 61 (58%) patients had polyclonal lymphocytic proliferation, of which lymphadenopathy and splenomegaly were the most frequent. Gastrointestinal disease was seen in 21 (20%) patients and 11 (10%) patients had granulomas. Other frequently occurring conditions were asthma (24%) and bronchiectasis (43%) (Figure 3). Malignancy was reported in 14% of “probable CVID” patients; these 16 malignancies in 15 patients included five non-Hodgkin lymphomas, three gastrointestinal cancers, three breast cancers, one large granular lymphocyte (LGL) leukemia, as well as single cases of myeloid sarcoma, melanoma, cervical, and renal carcinoma (Figure 4). Interestingly, altogether, four “probable CVID” patients had some form of LGL lymphoproliferation (LGL-L). Out of the defined clinical phenotypes, 21 (20%) “probable CVID” patients had only one, while the rest had up to all five phenotypes (Figure 5). Among the 26 “possible CVID” patients, five malignancies, each with a single case, were found: non-Hodgkin and Hodgkin lymphoma, cervix cancer, chronic myeloid leukemia, and lung cancer.

### Clinical Differences between Probable and Possible CVID Patients

The overall number of infections (Figure 2), recurrent HSV (5 vs 15%, *p* = 0.074), bronchiectasis (43 vs 27%, *p* = 0.180), asthma (24 vs 39%, *p* = 0.141), autoimmunity (51 vs 35%, *p* = 0.36), and malignancy (14 vs 19%, *p* = 0.55) were not different between “probable” and “possible CVID” patients, respectively. However, polyclonal lymphocytic proliferation (70 vs 35%, respectively, *p* = 0.001), especially splenomegaly (39 vs 12%, *p* = 0.016) and lymphadenopathy (37 vs 15%, *p* = 0.039), were more frequent in “probable CVID” (Figure 3). While infections were more common in “probable CVID” (98 vs 85%, *p* = 0.014), these patients also had more additional comorbidities. Therefore, there were more patients with infections only among “possible CVID” (36 vs 15%, *p* = 0.019) (Figure 5). All in all, patients with “probable CVID” could be divided into 16 clinical phenotypic combinations and “possible CVID” into 12 clinical phenotypic combinations. The dominant phenotypes covering altogether 67.9% of “probable CVID” patients were “infections only” and infections in combination with “autoimmunity” and/or “polyclonal lymphocytic proliferation” (Figure 5).

### Laboratory Differences between Probable and Possible CVID Patients

Chronic thrombocytopenia was more frequent in “probable” than “possible CVID” (33 vs 4%, *p* = 0.001), while chronic neutropenia was found similarly in both (16 vs 12%, *p* = 0.76). Of note, there was a trend, albeit statistically insignificant, toward more frequent chronic lymphopenia in “probable CVID” (44 vs 27%, *p* = 0.126). The numbers of CD19^+^ B and CD4^+^ T cells and the concentrations of immunoglobulin A, G, M, and E were lower in “probable” than in “possible CVID” patients (Table 4).

**Table 4:** Mean immunoglobulin plasma concentration and the lymphocyte subclasses in possible and probable CVID.

Group		IgG	IgM	IgA	IgE	CD19^+^	CD3^+^	CD4^+^	CD8^+^
Reference range		6.8–15	0.47–2.84	0.08–1.4	0–110	0.08–0.62	0.75–2.76	0.404–1.612	0.22–1.13
Unit		g/L	g/L	g/L	IU/L	×10^9^/L	×10^9^/L	×10^9^/L	×10^9^/L
Possible CVID	Mean	5.81	0.76	0.89	35.50	0.21	1.46	0.87	0.58
	SD	2.36	0.47	0.70	86.73	0.12	0.62	0.44	0.29
	*n*	26	26	26	26	26	26	26	26
Probable CVID	Mean	2.30**	0.32**	0.17**	4.56**	0.15*	1.35	0.63*	0.69
	SD	1.64	0.61	0.20	8.01	0.17	1.15	0.37	0.86
	*n*	106	106	106	99	103	103	104	103

Ig, immunoglobulin, given for each as a mean of plasma concentrations (gram per liters). Flowcytometry of lymphocytes was used to quantitate subclasses (number of cells ×10^9^/L). CD3: T-lymphocytes; CD19: B-lymphocytes, CD4: T-lymphocytes helper, CD8: T-lymphocytes suppressor. *n*, number of patients. Asterisks mark statistical significance between possible and probable CVID patients, *p < 0.01 and **p < 0.001 (Mann–Whitney *U*-test).

**Figure 6:**
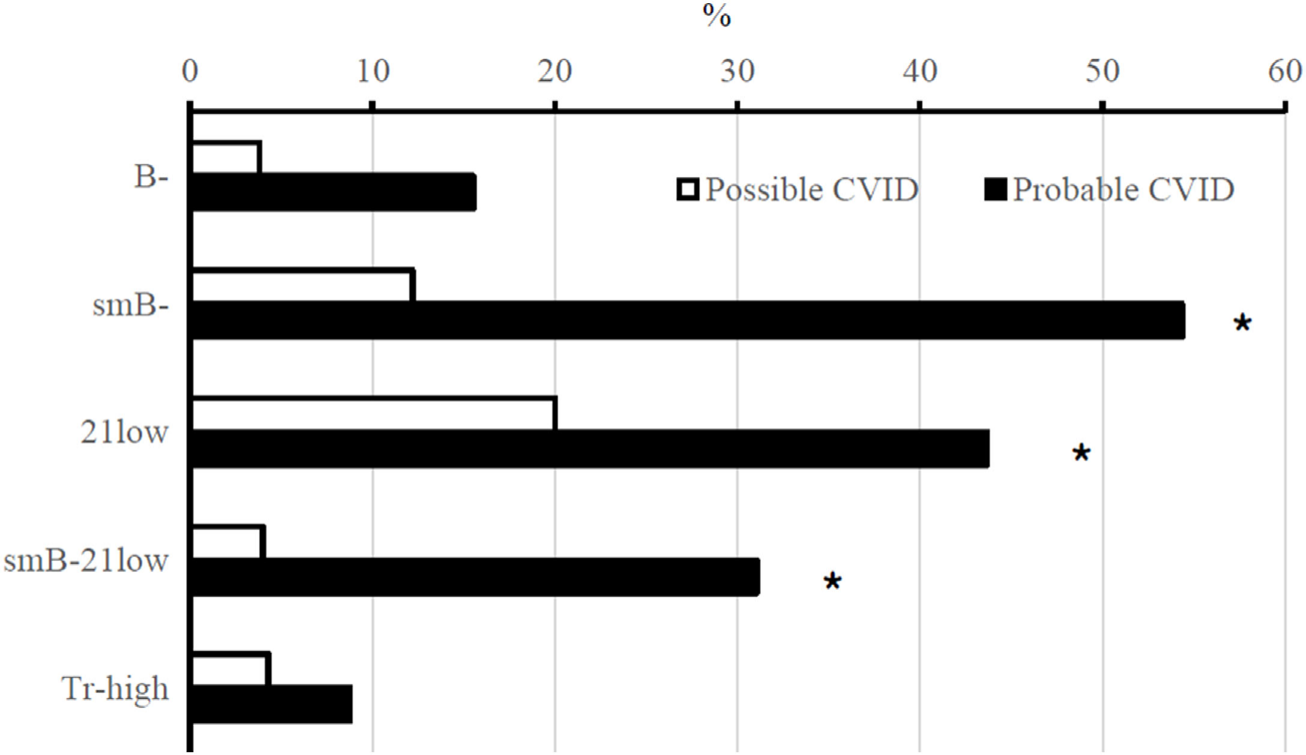
B-cell phenotyping results (%) in probable and possible CVID patients. Abbreviations: B^−^, CD19^+^ B cells ≤1% of lymphocytes; smB^−^, switched memory B cells ≤2% of B cells; 21low, CD21^low^ cells ≥10% of B cells; Tr-high, transitional B cells ≥9% of B cells (in total, 129 CVID patients had B-cell phenotyping data available. Transitional B cells had been studied in 114 subjects). Asterisks mark statistical significance between “probable” and “possible CVID” patients, *p < 0.01 (χ^2^-test).

In B cell phenotyping according to EUROclass, differences between “probable” and “possible CVID” were observed (20). The percentage of patients with CD19^+^ B cells <1% of all lymphocytes was 16% in “probable CVID” and 4% in “possible CVID” (*p* = 0.192). In patients with CD19^+^ B cells >1% of all lymphocytes, CD27, CD21, and IgM expression were analyzed. Class-switched memory B cells (CD27^+^IgM^−^) were <2% of CD19^+^ B lymphocytes in 62% of patients with “probable CVID” and in 12% of patients with “possible CVID” (*p* < 0.001). In “probable CVID,” 50% of patients had >10% of activated (CD38^low^CD21^low^) B cells vs 20% of patients in “possible CVID” (*p* = 0.007). Finally, only 4% of patients with “possible CVID” had, according to EUROclass, both low percentages of class-switched B cells and high percentages of activated B cells, while this was found in 36% of patients with “probable CVID” (*p* = 0.001) (Figure 6). Also, patients with GLILD had significantly lower class-switched memory B cells compared to those without GLILD (*p* < 0.001). Interestingly, when comparing B cell phenotypes and the risk of malignancy among all 132 CVID patients, 35.3% of patients with B cell loss, defined as <1% total lymphocytes being CD19^+^ B cells, had malignancies vs 12.2% of those without B cell loss (*p* = 0.024). CD16^+^ CD56^+^ NK cell counts did not differ between patients with or without malignancy. Low class-switched memory B cells were associated with autoimmunity (*p* = 0.025) and polyclonal lymphocytic proliferation (*p* < 0.001). Increased percentages of activated B cells according to EUROclass were associated with polyclonal lymphocytic proliferation (*p* < 0.001).

### Patients with Low IgG and Low IgM but Normal IgA

Unlike recently published diagnostic criteria, the ESID 2014 registry inclusion criteria suggest that patients with normal IgA but with low IgG and IgM should not be called CVID. We found three such patients in our “probable CVID” cohort. In two of them, the phenotype was “infections only.” One, however, had a typical, severe CVID phenotype with malignancy, autoimmunity, and infections. All three patients had class-switched memory B cells above 2% of B cells and normal CD21 expression. The lowest IgG in these patients averaged 4.2 g/L (3.9–4.5) vs average of 2.3 ± 1.6 g/L (±SD) in the “probable CVID” group as a whole.

## Discussion

Common variable immunodeficiency is the most common primary immunodeficiency in the world, with published prevalences ranging from 0.4 to 3.8/100,000 (Table 1) and an estimated prevalence ranging from 2/100,000 to 4/100,000 (21). However, our study showed a minimum adult prevalence of 6.9/100,000 (5.5/100,000 for “probable CVID”) in Finland (Table 2), the highest population prevalence ever reported. After a previous report’s prevalence of 2/100,000, this higher Finnish prevalence is likely due to improved diagnosis and awareness (16). Of the other Nordic countries, Denmark has the previously highest recorded prevalence of 3.8/100,000, while Iceland has 3.1/100,000 and Norway 2.1/100,000. In Central and Southern Europe, the prevalences vary from 1.3/100,000 in the United Kingdom to 0.6/100,000 in Spain (Table 1). We chose to exclude all patients with secondary causes to CVID. However, the diagnosis of primary vs secondary antibody deficiency can be very difficult in individual patients. Furthermore, patients with primary immunodeficiencies frequently receive immunosuppressive medication and display a combination of PID and secondary immunodeficiency.

The prevalence of CVID in the studied hospital districts varied from 1.5/100,000 in the easternmost Southern Finland to 7.7/100,000 in the greater Helsinki area (*p* = 0.006). However, due to the relatively small sizes of studied hospital districts outside greater Helsinki, even a single new patient would strongly influence the prevalence in those areas (Table 2). Prevalence differences may furthermore reflect lack of awareness for primary immunodeficiencies among local doctors and a need for improved screening and referral practices. Genetically, the population around Helsinki capital area originates from continuous migration over centuries from all regions of country and likely represents the entire Finnish population well. Thus, its adult prevalence of CVID of 7.7/100,000 (6.1 for “probable CVID”) further suggests a need for countrywide improvements in referral practices.

The observed differences between the three diagnostic criteria stemmed mainly from varying demands for diagnostic findings. Since ICON requires vaccine responses, it was the most restrictive in our partly historical cohort, while in vaccine response-studied patients, other assessed criteria seemed strictest. As there was disagreement for probable/definite CVID between different criteria in only eight patients (8%), most patients with typical CVID seemed unproblematic to diagnose. In practice, a much wider variation in setting a diagnosis of CVID between patient cohorts likely stems from different criteria used to define impaired vaccine responses (15, 23, 24). The poor specificity of previously promoted seroresponse criteria has recently been proven formally in the to date largest cohort of Belgian control subjects (23). Since only a subset of our patients’ antipneumococcal responses was studied with a similar bead-based multiplex assay as in the Belgian study, direct comparisons were rendered difficult. However, both suggested sets of reference cut-offs result in clearly smaller numbers of CVID patients than if the AAAAI criteria for vaccine responses were used (15, 23, 24).

Common variable immunodeficiency was equally common in men as in women. However, males presented at an earlier age than females, corroborating earlier studies (21). In older patients, the prevalence was higher among females. Whether this represents earlier onset in males or delayed diagnosis in females remains to be studied.

Comparing the phenotypes of Finnish CVID patients to those in other published cohorts, the phenotypes of CVID patients in Britain seem closest (25). Even though Western Finns and Swedes share a common genetic background, the phenotypes reported in Swedish CVID patients appear clearly different (25, 26). Most of our patients had multiple non-infectious complications. Even though most patients had a history of infections, only 23% had a clinical picture with infections only. Furthermore, 73% of our patients displayed more than one phenotype. All in all, our patients could be categorized into 18 different combined phenotypes (17). This was in stark contrast to a previous European study where 83% of the patients had only one phenotype (25).

Interestingly, though LGL-L has occasionally been described as a complication of CVID, it occurred during follow-up as frequently as in four of 132 patients (3%) in our cohort (27). One of the patients with LGL-L further developed LGL leukemia. LGL-L is a well-known phenomenon in, for example, *GATA2* and *STAT3* gain-of-function mutated patients, who may also mimick CVID phenotype (28, 29). Our CVID patients with LGL-L underwent whole exome sequencing and exclusion of GATA2 haploinsufficiency at the RNA level, without findings. Thus, our results confirm that LGL-L is a true CVID-associated complication and emphasize that primary immunodeficiency should be excluded in patients with LGL-L.

In general, “possible CVID” patients had fewer clinical phenotypes than those with “probable CVID” (Figure 5). “Possible CVID” patients presented with a single phenotype in 54% while this was seen only in 20% of “probable CVID” patients (*p* < 0.001, Fisher’s 2-sided exact test). This was especially true concerning infections and polyclonal lymphocytic proliferation. Also, B cell phenotypes were less frequently abnormal among “possible” than “probable CVID” patients. As reported previously by others (20), low class-switched memory B cells were associated with autoimmunity and lymphoproliferation. In “B cell loss,” B cell numbers were low at diagnosis and frequently lead to the disappearance of B cells in blood during follow-up. Interestingly, in our cohort, B cell loss seemed to be associated with malignancy, but numbers are small. Both B cell loss and propensity to malignancies could hypothetically reflect some uncharacterized genetic predisposition and requires further study. To the best of our knowledge, B cell loss also seems somewhat more common in Finland than in previously reported cohorts.

With the exception of the one previously published patient with low IgA, IgM, and an *NFKB1* I553M mutation (22), “possible CVID” patients displayed “IgG deficiency” (low IgG together with impaired antipneumococcal polysaccharide responses). Our “possible CVID” or “IgG deficiency” cohort was thus largely identical to a suggested group of patients with “idiopathic primary hypogammaglobulinemia,” and our findings largely corroborate earlier reports (22).

Our findings in three clinically typical “probable CVID” patients suggested that patients with normal plasma IgA levels, low IgG, low IgM, and impaired polysaccharide responses should, despite their rarity, also be called CVID. This became one of the reasons not to compare the ESID 2014 registry criteria to other criteria. However, we also agree with the notion that those with for example prolonged lymphocyte subclass cytopenias and low naïve CD3^+^CD4^+^ T cells or mitogen responses would most likely need a separate diagnostic class. Such patients may represent hitherto uncharacterized combined immunodeficiencies.

A major weakness in our study was its retrospective nature. Also, the lack of systematic assessment for T cell deficiency in our historical cohort should be regarded as a further weakness, only partly offset by the long follow-up periods in such patients. Further, due to the lack of systematically collected registry data, we could not assess the prevalence of CVID in patients ≤16 years of age at the end of the study; the true prevalence of CVID in Finland is thus likely even higher than the 7.7/100,000 found in the greater Helsinki area. However, all pediatric CVID patients in the study area are systematically referred to the study units at age ≥16; our available results thus suggest that pediatric-onset of CVID seems surprisingly rare in Finns (Figure 1). We are currently entering both pediatric and adult primary immunodeficiency patients into a dedicated registry within our electronic patient record system.

Whole exome sequencing has been carried out on 51 patients in our cohort (data not shown). In them, only three unpublished patients with known disease-associated *TNFRSF13B* risk mutations and two published patients with a disease-causing *NFKB1* I553M mutation were found (22). Our prevalence results thus suggest an autosomal recessive condition and a founder effect of yet unknown gene(s) (26, 30). The high prevalence of CVID in Finland may be explained partly by Scandinavian genetic influence and partly, like in pathogenic *AICDA* M139T mutations, by the so-called Finnish Disease Heritage. This is characterized by enrichment of rare mutation(s) in a genetically isolated population (26, 30–32). The genetic background in Finland generally correlates with geography. The Eastern population is a well-defined founder population, with later migration to Northern Finland (26, 30–32). Whether the high prevalence of CVID in the Helsinki area stems from the Eastern, Western, or both subpopulations, remains to be studied. If the observed high prevalence indeed genetically also stems from the Eastern genetic isolate, increased awareness in primary care and remittal for assessment will be especially needed in Northern and Eastern Finland.

To conclude, we report an unprecedentedly high (adult) prevalence of CVID from Finland. Our results suggest unknown disease causing gene(s) in the Finnish CVID population, a known genetic isolate (26, 30–32). In the future, Finland may become an optimal country to study the etiology of CVID due to suspected founder effects, a limited amount of background genetic variation, access to hospital and registry data covering the whole country, an advanced biobank law and robust research infrastructure. Our goal will be to search for gene candidates in the known familiar and geographic clusters of CVID in Finland. Recognizing patients with CVID earlier improves patient outcomes and the quality and cost efficiency of care (33). Thus, raised awareness about CVID especially among primary and secondary care physicians in Finland will be needed to facilitate diagnosis in areas of Finland with low reported prevalence.

## Ethics Statement

This study was carried out in accordance with the Finnish Law on Medical Research (488/1999) and the recommendations of Coordinating Ethics Committee, Hospital District of Helsinki and Uusimaa with written informed consent in accordance with the Declaration of Helsinki. The protocol was approved by the Coordinating Ethics Committee, Hospital District of Helsinki and Uusimaa (138/13/03/00/2013).

## Author Contributions

TM, JK, MF, and MS designed the study, coordinated the project, and participated in writing the article. JSe and SP collected the data and participated in statistical analyses and writing. TM and SP participated in statistical analyses. SS contributed to writing the article and performed laboratory analyses. EE and JSa aided in data collection and performed genetic studies. TM, EM, AA, RP, PS, SP, AJ, and MS aided in data collection and provided clinical care for the patients. All authors have read and approved the final manuscript.

## Conflict of Interest Statement

MS has received honoraria from CSL Behring and Octapharma, TM from CSL Behring, and JSa from Roche and Merck. The remaining authors declare no competing financial interests.
